# Uterine Rupture on MRI Presenting as Nonspecific Abdominal Pain in a Primigravid Patient with 28-Week Twins Resulting in Normal Neurodevelopmental Outcomes at Age Two

**DOI:** 10.1155/2019/2890104

**Published:** 2019-07-16

**Authors:** Kathryn L. Ponder, Rosa Won, Laurel Clymer

**Affiliations:** John Muir Medical Center, Walnut Creek, CA 94598, USA

## Abstract

**Background:**

Uterine rupture is a rare occurrence that requires a high index of suspicion, particularly in a primigravid patient who presents prior to the onset of labor. Mortality rates are particularly high in primigravid patients.

**Case:**

A 36-year-old gravida 1, para 0 patient with dichorionic diamniotic twins presented at 28-weeks of gestation with abdominal pain. The pain was initially intermittent and felt to be musculoskeletal in origin. Ultrasound imaging after 3 days of worsening abdominal pain revealed extrauterine fluid, prompting an urgent MRI. MRI diagnosed the uterine rupture with hemoperitoneum and herniation of both amniotic sacs outside of the uterus, including one twin's torso and extremities, prompting emergency cesarean section. The premature twins required 2-month hospitalizations and had no neurodevelopmental impairments at 2-year follow-up.

**Conclusion:**

We present a unique case of rupture of an unscarred uterus in a primigravid patient prior to the onset of labor. Multiple gestation is a risk factor. This report adds to a handful of cases in which a history of endometriosis or extrauterine pelvic surgery was also present. The use of ultrasound and MRI to evaluate nonspecific abdominal pain led to the diagnosis and survival of both the mother and her premature twins.

## 1. Introduction

Uterine rupture is a rare occurrence (3-5.9 per 10,000 pregnancies) that requires a high index of suspicion, particularly in a primigravid patient who presents with nonspecific symptoms prior to the onset of labor [1, 2]. Here we discuss a primigravid patient with a history of endometriosis and extrauterine pelvic surgery presenting with abdominal pain in the setting of a dichorionic diamniotic twin pregnancy at 28 weeks of gestation. The patient's nonspecific symptoms and normal laboratory studies may have led to an initial delay in diagnosis; however, persistent symptoms and ultrasound imaging revealed a clue of extrauterine fluid, which prompted a stat MRI, diagnosis, and emergent intervention. This case demonstrates a rare presentation, occurring in an unscarred uterus and prior to the onset of labor. Between 3.1 and 12.9% of uterine ruptures in developed countries occur in an unscarred uterus (i.e., no history of cesarean section or hysterotomy) [2, 3]. Spontaneous uterine rupture in an unscarred uterus that occurs prior to the onset of labor is even rarer, representing only 13.6-16% of spontaneous cases [2, 4] and representing only 1.9% (4 out of 208 cases) [2] to 6.4% (3 out of 47 cases) [4] of all cases of uterine rupture. Maternal and perinatal mortality rates are particularly high in primigravid patients with spontaneous rupture compared to multigravid patients with a history of cesarean section [1–8].

Despite herniation of both intact amniotic sacs including the body of one twin into the peritoneal cavity, the patient and the resulting 28-week infants survived and did well. The infants were discharged after a 2-month hospitalization. They were evaluated in the High Risk Infant Follow-Up Clinic after discharge and had no neurodevelopmental impairments on the Bayley Scales of Infant and Toddler Development (Third Edition) and medical evaluation at 2 years of age. In this case, a high index of suspicion and the use of ultrasound and MRI to evaluate nonspecific abdominal pain resulted in the unique outcome of normal neurodevelopment in premature twins after uterine rupture.

## 2. Case Presentation

The patient was a 36-year-old U.S.-born woman of Indian ethnicity and high education level with a past medical history notable for endometriosis. Her surgical history was notable for 2 pelvic surgeries in the 2 years prior to her pregnancy. First she underwent removal of a large ovarian cyst, which began as a laparoscopy but converted to a laparotomy due to intestinal adhesions to the ovary and bleeding. The second procedure was a laparoscopic tubal ligation in the setting of hydrosalpinx that was uncomplicated. Sites of endometriosis and intestinal adhesions were noted intraoperatively but were not lysed at that time. She had no history of uterine surgery or dilation and curettage. Dichorionic diamniotic twins were conceived via in vitro fertilization (IVF), which involved uterine manipulation to the extent of an embryo transfer. At 13 weeks of gestation, she had a pulmonary embolus for which she was on enoxaparin sodium 80 mg subcutaneously twice a day. She had normal prenatal labs.

She was a gravida 1, para 0, at 28 weeks of gestation at the time of presentation. Three days prior to delivery she noted the new onset of left-sided abdominal pain described as intense “pressure.” This initially subsided and then recurred the following day. One day prior to delivery she presented to triage of the Labor and Delivery floor with a chief complaint of worsening left-sided abdominal pain. She rated the pain as 9 out of 10 and described it as “sharp, shooting, spasm” in nature, from the left side of her rib cage down to her hip. The pain improved after a dose of hydrocodone-acetaminophen. She rated her pain as 6 out of 10 about 1 hour later. Complete blood count (CBC), electrolytes, and an AmniSure test were evaluated. The CBC showed a white blood cell count of 16.8 × 10^∧^3/*μ*L (81% neutrophils, 14.7% lymphocytes), hemoglobin 10.4 g/dL, hematocrit 31.4%, and platelet count 215,000/*μ*L. Her glucose was 129 and albumin 2.7 with an otherwise normal electrolyte panel and liver function tests. A point of care AmniSure test for rupture of membranes was negative for amniotic fluid. Her other prenatal labs were all previously normal and she had no history of drug abuse. Vital signs were as follows: temperature 36.6°C, mild tachycardia with a heart rate of 120 beats per minute, respiratory rate 18-20, blood pressure 119/59, and SpO2 96-100%. The pain was felt to be most likely musculoskeletal in origin. She was sent home with a plan to follow up in the perinatologist's clinic the following morning at an appointment that had been previously scheduled.

In the perinatologist's office the following morning, she noted worsening abdominal pain. An ultrasound was performed, which showed an extrauterine fluid collection in the peritoneal cavity. She was given a dose of betamethasone and sent to the emergency department for a stat MRI of the abdomen and pelvis. In the emergency department her exam was notable for abdominal tenderness to palpation and guarding. She was otherwise alert and oriented with the following vital signs: temperature 37°C, heart rate 127 beats per minute, respiratory rate 20, and blood pressure 123/81. Fetal heart tracings were noted to be normal for age with moderate variability. No uterine contractions were present. She received a dose of IV morphine and IV fluids.

**Figure 1:**
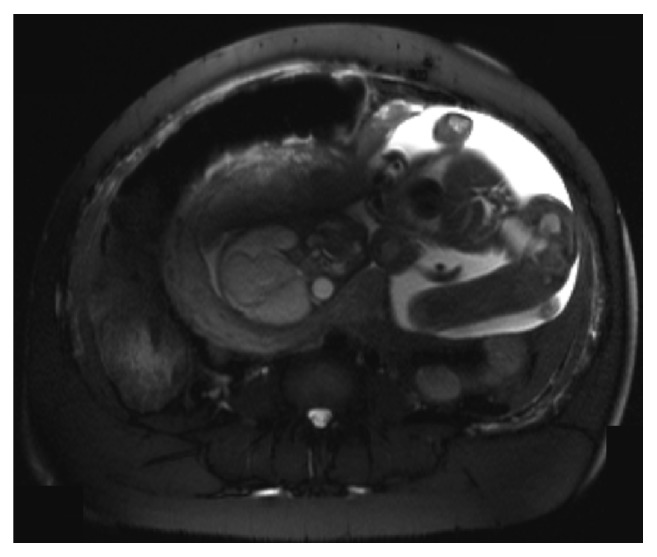
Lower portion of one fetus protruding out of the posterior aspect of the uterine fundus into the peritoneal cavity.

MRI of the abdomen and pelvis revealed a large defect within the left uterine wall with herniation of the body and extremities of one of the twins through the herniated defect. The head remained within the uterine cavity. A large portion of the intact amniotic sac of the second twin was also herniated through the uterine defect. There was moderate hemoperitoneum (Figure 1). Blood products were ordered in preparation for cesarean section. Her hemoglobin and hematocrit had decreased to 9 g/dL and 27.9% at the time of admission to the Labor and Delivery floor and 2 hours later, just prior to surgery, had decreased further to 8.2 g/dL and 25.1%.

### 2.1. Intervention

After imaging confirmation, the patient underwent emergency exploratory laparotomy and cesarean section through the uterine rupture at 28 weeks and 3 days of gestation. She received a 4 gram magnesium sulfate bolus for fetal neuroprotection and a dose of cefazolin at the start of the cesarean section. Intraoperatively, when the omentum was moved out of the way, an old blood clot was noted in the left upper side of the abdomen. The blood clot was removed and the amniotic sac was seen protruding into the peritoneal cavity from the site of the uterine rupture which was left lateral and cornual. The amniotic sac was ruptured and clear fluid was noted. The other twin's membrane was then seen and ruptured, again with clear fluid and delivered breech without difficulty, as the obstetrics team felt she could be delivered most quickly. Her cord was milked for autotransfusion of cord blood, clamped, and handed to the neonatology team. The second twin was immediately felt for. Her feet were grasped, the baby was delivered to the level of both scapulae, and the arms were sequentially swept across the chest. The fetal head was delivered via the Mauriceau–Smellie–Veit maneuver. The cord was milked for autotransfusion of cord blood and clamped, and she was handed to the awaiting neonatal resuscitation team. The placenta was delivered via massage. Pitocin was added to the IV fluids once the placenta was delivered. The uterus was cleared of all clots and debris with a moist laparotomy sponge. The uterine rupture site was closed with #1 Chromic suture in running-locked fashion. A second layer was also used to close the uterus with #1-0 Chromic suture in an imbricating fashion. One additional suture was required for another layer of closure. Two box stitches were needed for hemostasis. Excellent hemostasis was noted. There were adhesions of the bowel and bladder to the uterus which were not lysed given the complexity of the adhesions. The gutter and peritoneal cavity were copiously irrigated. Floseal was applied to the uterine closure. There was an estimated blood loss of 1000 mL during the procedure. The patient received 1000 mL of intravenous fluid and a unit of fresh frozen plasma intraoperatively.

### 2.2. Maternal Outcome

The patient remained in stable condition postoperatively. The postoperative course was complicated by anemia: several hours later on post-op day #1, her hemoglobin was as low as 6.3 g/dL, with a hematocrit of 19.3%, for which she received 2 units of packed red blood cells with normalization thereafter. She was placed on unfractionated heparin postoperatively due to her history of pulmonary embolism. She was discharged on postoperative day #3 on enoxaparin sodium 80 mg twice a day. Outpatient genetics consultation was obtained. Sequencing and deletion/duplication analysis of the COL3A1 gene was sent to evaluate for vascular Ehlers-Danlos Syndrome (type IV), which is associated with organ rupture, and was negative. Ultimately it was felt that the twin gestation may have placed her at higher risk as opposed to an underlying genetic condition.

### 2.3. Twin A Neonatal Course and Outcome

Twin A was a female with a birthweight of 1140 grams. She required 4 minutes of positive pressure ventilation (PPV) and then transitioned to Continuous Positive Airway Pressure (CPAP) with an Apgar score of 2 at 1 minute and 8 at 5 minutes. A capillary blood gas around 1 hour of life had a pH 7.31/pCO2 38.1/bicarb 18.7/ base deficit -6.4. She had Respiratory Distress Syndrome (RDS) and required nasal respiratory support for 2 weeks, but did not require oxygen support after that time. She was treated with caffeine for apnea of prematurity. A blood culture drawn at admission remained negative. She had a very small patent ductus arteriosus (PDA) on echocardiogram that remained until discharge. Head ultrasounds on day of life 9 and at 6 weeks of age were normal for age. She passed her newborn hearing screen. Her retinas were immature at the time of discharge, and noted to be mature at outpatient follow-up with no Retinopathy of Prematurity (ROP). She was hospitalized for 56 days and discharged at 36 weeks and 2 days adjusted age.

After her Neonatal Intensive Care Unit (NICU) discharge, she followed up with the cardiology clinic. A tiny PDA was still present on echocardiogram at 1 year of age, which did not require surgical correction. She followed up with the ophthalmology clinic annually for routine evaluations with normal vision. At 19 months adjusted age (21 months chronological age) she demonstrated typical development on the Clinical Linguistic and Auditory Milestone Scale (CLAMS) and the Cognitive Adaptive Test (CAT) of the Capute Scales. Her fine and gross motor skills were in a typical range for her adjusted age. At 26 months of age she was evaluated with the Bayley Scales of Infant and Toddler Development (Third Edition). She demonstrated “Above Average” Scaled Scores on the cognitive, expressive language, and receptive language portions and “Average” Scaled Scores for both gross motor and fine motor skills. Her Composite Scores were classified as “Superior” for cognition, “High Average” for language, and “Average” for motor skills (Table 1).

### 2.4. Twin B Neonatal Course and Outcome

Twin B was a female with a birthweight of 1115 grams. She required 7 minutes of PPV and then transitioned to CPAP in the delivery room with Apgar scores of 3 at 1 minute and 7 at 5 minutes. A capillary blood gas at 1 hour of life had a pH 7.33/pCO2 38/bicarb 19.7/base deficit -5.2. Her neonatal course was complicated by RDS, apnea of prematurity, and supraventricular tachycardia (SVT). She required intubation for 1 day with surfactant administration for RDS and then nasal respiratory support for 2 weeks. She was treated with caffeine for apnea of prematurity. A blood culture drawn at admission remained negative. She had 2 episodes of A-V reentrant SVT in the first week of life and another at 4 weeks of life, prompting cardiology consultation and digoxin treatment with no recurrent episodes. A head ultrasound on day of life 9 showed a tiny left choroid plexus cyst, a normal variant. A screening head ultrasound at 8 weeks of age showed resolution of the left choroid plexus cyst and new right choroid plexus cysts, also thought to be normal variants. There was no intraventricular or parenchymal hemorrhage and there was normal appearing periventricular white matter, consistent with a normal head ultrasound for her age. She passed her newborn hearing screen. Her retinas were immature at the time of discharge and noted to be mature at outpatient follow-up with no ROP. She was hospitalized for 60 days and discharged at 36 weeks and 6 days adjusted age.

After her NICU discharge, she followed up with the cardiology clinic with no recurrence of SVT. Digoxin was discontinued at 6 months of age. A small, hemodynamically insignificant PDA was still present but did not require surgical intervention. She followed up with the ophthalmology clinic annually for routine evaluations with normal vision. She followed up with feeding therapists and the gastroenterology clinic due to difficulty with feeding (emesis, oral aversion, and delayed gastric emptying). She was able to support her nutrition and growth with oral feedings and did not require a post-discharge feeding tube. Symptoms were improved by 2 years of age. At 19 months adjusted age (21 months chronological age) she demonstrated typical development on the CLAMS and the CAT of the Capute Scales. Her fine and gross motor skills were also in a typical range for her adjusted age. At 27 months of age she was evaluated with the Bayley Scales of Infant and Toddler Development (Third Edition). She demonstrated “Above Average” Scaled Scores on the cognitive, expressive language, receptive language, and fine motor portions and an “Average” Scaled Score for gross motor skills. Her Composite Scores were classified as “Superior” for cognition, “Very Superior” for language, and “High Average” for motor skills (Table 1).

## 3. Discussion

There are 3 major categories of uterine rupture: (1) presence of a uterine scar (history of cesarean section or hysterotomy), (2) trauma to an unscarred uterus (breech extraction, forceps, oxytocin, fundal pressure, and shoulder dystocia), and (3) spontaneous rupture of an unscarred uterus [4]. Most cases of uterine rupture in developed countries fall into the first category, which occur in the setting of a uterine scar. Only 3.1-12.9% of cases in developed countries fall into the second and third categories, which occur in the setting of an unscarred, intact uterus [2, 3]. This is in contrast to countries characterized as “less and least developed” by the United Nations, where most cases occur in an unscarred uterus [1]. Cases may be further subdivided based on whether they occur before or after the onset of labor. Uterine rupture that occurs both spontaneously and prior to the onset of labor is very rare, contributing to only 1.9-6.4% of uterine rupture cases overall [2, 4].

While both are rare, the spontaneous and traumatic types involving an unscarred uterus are associated with more catastrophic outcomes as far as maternal and perinatal morbidity and mortality [1, 2, 4]. Despite its rarity, uterine rupture accounts for about 5% of maternal deaths in the United States [4]. The risks of maternal hemorrhage requiring transfusion, intensive care unit admission, and hysterectomy are higher with an unscarred uterine rupture as compared to a uterine scar rupture [2]. Perinatal mortality rates associated with uterine rupture in less and least developed countries range from 74 to 92% [1], whereas rates of 8.7 to 11.7% are reported for developed countries [2, 3]. The risk of perinatal death is 3.8 times higher in the setting of an unscarred uterine rupture compared to a uterine scar rupture [2]. Population-based studies in more developed countries estimate that the incidence of uterine rupture is about 3 to 5.9 per 10,000 deliveries [1, 2]. A population-based study in the Netherlands reports an incidence of 5.1 per 10,000 pregnancies in women with a uterine scar [2]. For women without a uterine scar, the incidence is as low as 0.6 to 0.7 per 10,000 pregnancies [2, 9].

Besides a prior cesarean section, Zwart and colleagues note an increased risk for uterine rupture in women with epidural anesthesia, induction of labor, postterm pregnancy, non-Western immigrants, and advanced age [2]. In addition, other reports identify multiparity (particularly grand multiparity), placenta percreta, placental anomalies, prior uterine instrumentation, malpresentation, fetopelvic disproportion/macrosomia, version procedures, uterine anomalies, and history of gestational trophoblastic disease as risk factors to be considered [4, 9–12].

Multiple gestation, including twins, is a risk factor for uterine rupture which has been identified in numerous studies, as with this case [13, 14]. It is possible that this patient's previous extrauterine pelvic surgeries or endometriosis could have also contributed to the rupture. One other report indicates that out of 25 cases of rupture of an unscarred uterus, n = 1 had a history of another pelvic surgery and n = 2 had a history of endometriosis, as with the case we present [2]. Other possible contributing factors in the remaining cases in that report include a history of uterine instrumentation or manipulation (instrumental abortion, postpartum curettage, and hysteroscopy), history of ectopic pregnancy, and uterine fibroids. In 52% of cases with an unscarred uterus (n = 13), however, the authors do not identify any risk factors. The study does not include genetic testing for vascular Ehlers-Danlos Syndrome (type IV), a rare condition associated with organ rupture [15]. Analysis of the COL3A1 gene was negative for vascular Ehlers-Danlos Syndrome in our patient. The patient we present had a history of uterine manipulation to the extent of embryo transfer for IVF, but this has not been associated with rupture in any reported cases to our knowledge.

Other rare cases of spontaneous prelabor rupture of an unscarred uterus have been reported. These cases have included more catastrophic outcomes such as hysterectomy with perinatal mortality [6, 7] or both maternal and perinatal mortality [8]. The very preterm gestational age of the twins in this report adds to the rarity of the positive outcome. We identified only one other case of prolonged partial extrusion of a fetus from an unscarred ruptured uterus prior to labor with a good outcome from 1982, which was a singleton gestation [16]. Interestingly this occurred at a hospital located 15 miles away from our center. We did not identify any other reports with 2-year neurodevelopmental follow-up.

MRI imaging is an important tool in the diagnosis of uterine rupture, particularly in the setting of nonspecific abdominal pain in a patient with any of the previously mentioned risk factors. Advantages of MRI include the ability to visualize the uterine wall defect for a more definitive diagnosis, less operator dependence and less discomfort for a patient with abdominal pain compared to ultrasound, and no ionizing radiation with better visualization of soft tissues compared to CT scan [17]. Thus far, few reports aside from the case we have presented have described the associated MRI findings [17, 18].

## 4. Conclusion

We present a unique case of rupture of an unscarred uterus in a primigravid patient prior to the onset of labor. Multiple gestation is an important risk factor for uterine rupture. This report adds to a handful of other cases in which a history of endometriosis or extrauterine pelvic surgery was noted prior to an unscarred uterine rupture. A high index of suspicion and the use of ultrasound and MRI to evaluate nonspecific abdominal pain led to the diagnosis and survival of both the mother and her premature twins, with no neurodevelopmental impairments at 2 years of age.

## Figures and Tables

**Table 1:** Bayley Scales of Infant and Toddler Development (Third Edition) Composite Scores at 2-year follow-up. Composite Scores classify whether the child's overall development is within the average range. The average range is considered to be 90 to 109.

	Subtest	Score	Range	Classification
Twin A				

	Cognitive	120	110-126	Superior
	Language	118	110-124	High Average
	Motor	103	95-110	Average

Twin B				

	Cognitive	120	110-126	Superior
	Language	138	128-143	Very Superior
	Motor	115	106-121	High Average
